# Granulomatosis with Polyangiitis (GPA)—A Multidisciplinary Approach of a Case Report

**DOI:** 10.3390/medicina58121837

**Published:** 2022-12-13

**Authors:** Cornelia M. Trandafir, Nicolae Constantin Balica, Delia I. Horhat, Ion C. Mot, Cristian A. Sarau, Marioara Poenaru

**Affiliations:** 1 Department of ENT, Victor Babeş University of Medicine and Pharmacy, 300041 Timisoara, Romania; 2 ENT Department, SCMUT Hospital Timisoara, Bd. Revolutiei No. 6, 300054 Timisoara, Romania; 3 Department of Medical Semiology I, Victor Babeş University of Medicine and Pharmacy, 300041 Timisoara, Romania

**Keywords:** otorhinolaryngology, granulomatosis with polyangiitis, interdisciplinary, vasculitis, ocular manifestations, diagnosis, treatment

## Abstract

Granulomatosis with polyangiitis is an atypical, multisystem disease with unknown etiology that generally affects both genders equally, with a predominance in the Caucasian racial group for individuals in their fourth decade. The disease affects the small vessels of the respiratory system, lungs, and kidneys. ENT manifestations are common, but ocular involvement is also frequent and can occur as an initial harbinger of the disease. The signs and symptoms of the disease are non-pathognomonic and sometimes localized, but it carries a poor prognosis if left untreated. Early diagnosis of granulomatosis with polyangiitis can be difficult and is established by a clinical examination along with laboratory tests for anti-neutrophil cytoplasmic antibodies (ANCA) and anatomopathological exam results that showcase necrosis, granulomatous inflammation, and vasculitis. Although the ocular involvement is not life threatening, it can cause blindness and may also be a sign of the active form of this systemic fatal disease. Treatment strategies involving immunosuppression and adjuvant therapies improve the prognosis. In this article we present a rare case of a patient diagnosed with granulomatosis with polyangiitis in our ENT department in 2003, with a follow-up for19 years in our clinic.

## 1. Introduction

Granulomatosis with polyangiitis (GPA) is a rare, immunologically mediated vasculitis associated with anti-neutrophil cytoplasmic antibodies (ANCA) [1]. It was first described in 1897 by McBride, with a further description in 1931 by Klinger, as a variant of polyarteritis nodosa, followed by Wegener in 1936 who characterized it for the first time as an individual, distinct syndrome, clinically and pathologically distinct from polyarteritis nodosa. 

Its annual incidence is reported to be between 5 and 10 cases/1,000,000 population [2] and rarely seen in children and young adults. Remission occurs in 85–90% of treated patients [3]. The relapse rate within 5 years is 50% [4]. The risk of mortality is secondary to subglottic stenosis, rapidly progressing glomerulonephritis, or respiratory system involvement. 

Although its etiology remains largely unknown, a few theories linked to genetic predisposition, environmental triggers, and the implication of infectious agents have been suggested [1,5]. 

The pathology is characterized by the development of a general, necrotizing, systemic vasculitis and a granulomatous inflammation pattern within the vessel wall and subendothelial space [6]. It usually affects the respiratory tract and kidneys; for that reason the acronym ELK (E—ear, nose and throat involvement; L—lung involvement; K—kidney involvement) is classically used. 

Goldman and Churg proposed in 1954 a useful criteria to diagnose the condition: (1) the presence of granuloma in the upper airways; (2) necrotizing vasculitis; (3) glomerulonephritis [7]. 

GPA is traditionally considered a disease with a predilection for renal and pulmonary involvement. 

Clinically, the patient complains of a wide spectrum of manifestations. They may present limited forms, involving one or two ELK areas, or a severe, generalized form due to the affliction of multiple organ systems (fever, acute pain, severe malaise, weakness) if left untreated. Renal involvement can be suspected if the patient has hematuria, proteinuria, or a cellular cast on the urine cytology, and can be manifested as acute kidney injury, chronic kidney injury, or renal failure [8,9]. 

An otolaryngologist plays an important role in its diagnosis and treatment [10]. The most common ENT manifestations include epistaxis, sinus inflammation, nasal obstruction, facial nerve palsy [11], and hearing loss [12]. Clinically, the literature reports septal perforation as the most common feature of damage and a prevalence of subglottic stenosis between 6 and 23% [13,14]. Other signs, such as mucosal ulcerations of the nose with bone and cartilage destruction, might be present with evolution of the disease. 

Granulomatosis with polyangiitis can also have pulmonary [15], renal, cutaneous, cardiovascular, and neurological manifestations. GPA can affect any part of the eye, and orbital involvement may be the first and only sign of the disease. The ophthalmological symptomatology is also nonspecific, but the anterior segment of the eye and the orbit are usually involved, consisting of episcleritis, scleritis, conjunctivitis, blindness, and nasolacrimal obstruction. According to Pakrou et al., ophthalmic involvement can result in significant morbidity and even blindness [16]. A remarkable association between nasolacrimal obstruction and subglottic stenosis was reported in the literature by Robinson et al. [17]. 

Laboratory tests show elevated levels of anti-neutrophil cytoplasmic antibodies (ANCA) with a cytoplasmic staining pattern directed against proteinase 3 (PR3). Being related to disease activity, ANCAs have been identified as risk factors of GPA relapse and are currently used in the long-term follow-up process [18]. 

As the symptomatology is nonspecific, the exclusion of other granulomatous diseases (bacterial, viral, fungal) and other diseases with unspecified etiology diagnosis is usually needed. 

Histologically, evidence of necrotizing granulomas usually indicates the diagnosis; however, treatment can be initiated even if a histological diagnosis cannot be made, if the clinical criteria of diagnosis are present and the c-ANCA titer is positive. 

The treatment of GPA is through medication and is based on a combination of immunosuppressants divided into various phases, followed by a maintenance treatment once remission has been achieved. 

## 2. Case Report

Female patient, 43 years old, previously diagnosed with granulomatosis with polyangiitis in our department in 2003 with a nasal and laryngeal determination, was admitted to the clinic for clinical-biological reassessment. 

From her medical history, we know that she suffers from: granulomatosis with polyangiitis with ANCA–PR3 positive in remission, chronic secondary glomerulonephritis in remission, asthma, secondary hypertension, hypokalemia, mild secondary anemia, mixed dyslipidemia with triglyceride predominance, secondary hyperparathyroidism, septal perforation, nasal granuloma, subglottic stenosis, and hearing loss after repeated otitis for which she had tympanotomy and auditory prosthesis. 

In light of her renal involvement (chronic secondary glomerulonephritis), she was treated by the nephrology department with a combination of corticoterapic treatment (prednisone) and cyclophosphamide. She was in remission under this pathognomonic treatment. 

**Figure 1:**
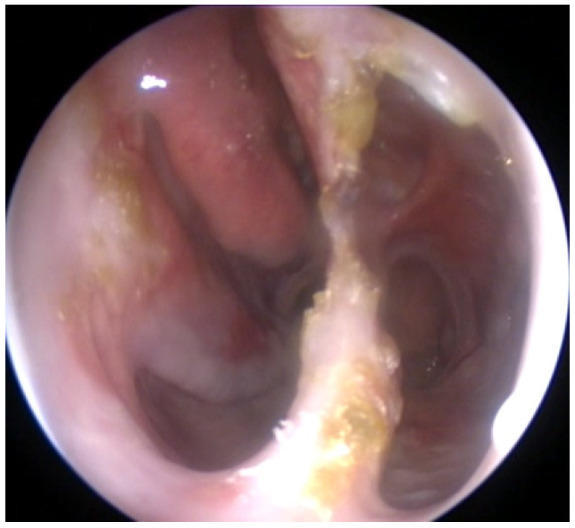
Nasal endoscopy.

The nasal endoscopy revealed a single cavity by disappearance of the septal cartilage, an atrophic, friable, crust-covered mucosa, almost complete disappearance of the inferior and middle nasal turbinate, and rhino pharynx covered by gray crusts (Figure 1). 

**Figure 2:**
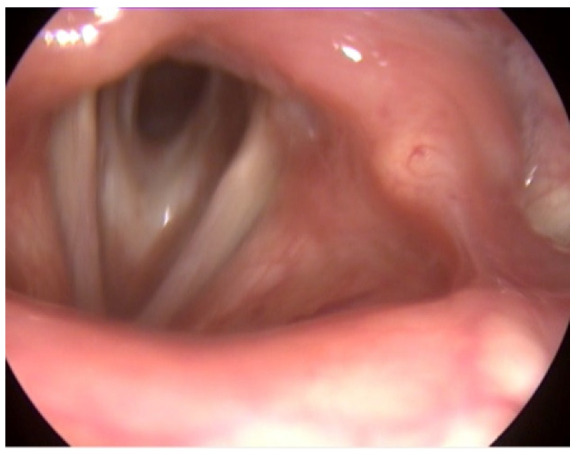
Subglottic stenosis.

The laryngeal endoscopy revealed subglottic stenosis and free vocal cords, which were mobile with breathing and phonation. The stenosis had not progressed since the last follow-up (Figure 2). 

**Figure 3:**
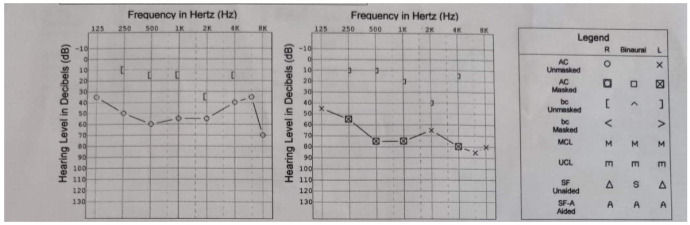
Hearing exam using an Itera II audiometer.

The audiogram showed mixed hearing loss after repeated otitis for which she had tympanotomy (Figure 3).

Ocular examination
BCVA (best corrected visual acuity) RE (right eye) = 20/25 (+1.00 dsf × −0.50 dcyl 10°)
BCVA LE (left eye) = 20/25 (+1.50 dsf × −0.50 dcyl 5°)
Normal ocular adnexa, normal eye motility.

**Figure 4:**
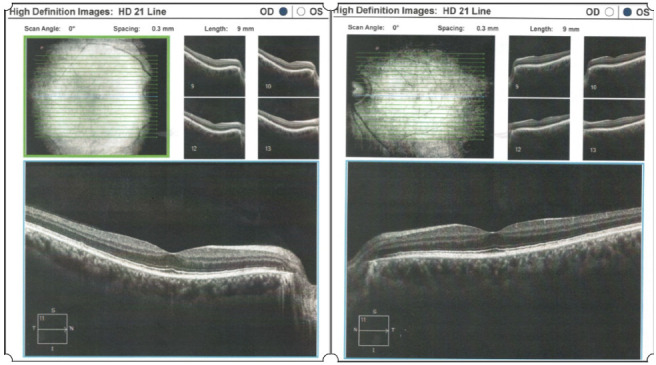
Optical coherence tomography.

**Figure 5:**
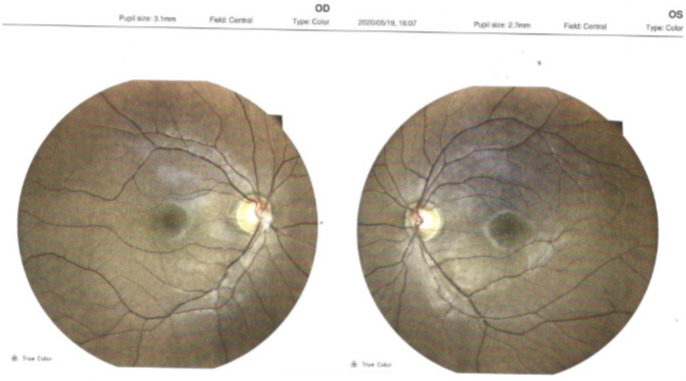
Retino photography.

**Figure 6:**
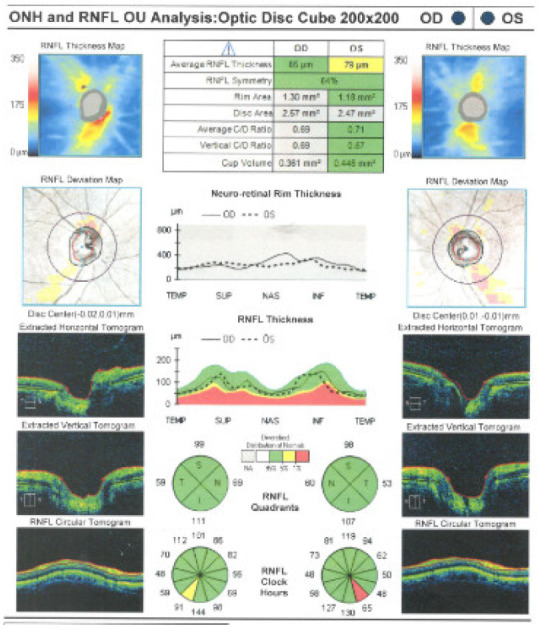
ONH and RNFL analyses.

**Figure 7:**
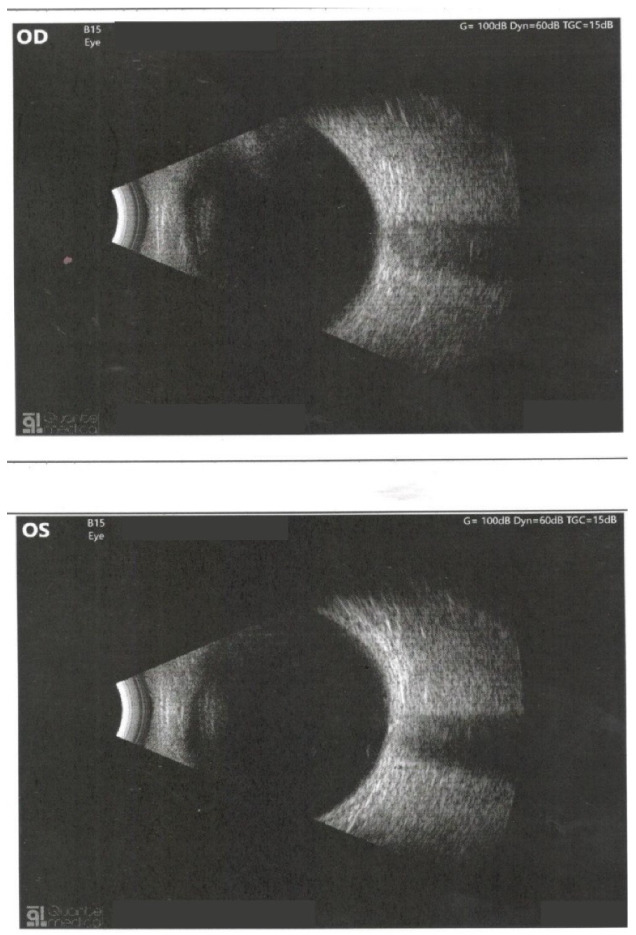
Ocular ultrasound.

The slit lamp examination revealed a normal aspect of the anterior pole. In order to correctly evaluate the posterior pole structures, a complex examination was performed that included: retino photography, ocular ultrasound, and optical coherence tomography (OCT) (Figure 4, Figure 5, Figure 6 and Figure 7). The pathological findings highlighted by the ancillary tests were: discreet miliary drusen, a slight reduction of the thickness of the retinal nerve fiber layer in the LE, and a C/D ratio of 0.69/0.7. These pathological features are not specific to granulomatosis with polyangiitis. 

## 3. Discussion

With an unknown etiology, granulomatosis with polyangiitis is a rare systemic disease characterized by granulomatous vasculitis of the small to medium sized vessels in almost any organ system [19], but it is usually seen in the upper respiratory tract and kidneys. The most common renal involvement is rapidly progressing, necrotizing glomerulonephritis [20]. GPA is associated with anti-neutrophil cytoplasmic antibodies (ANCA) [21]. 

It affects both genders equally in their fourth and fifth decades of life [22]. A prompt diagnosis and early treatment can reduce the morbidity and mortality of the disease. 

Up to 85% of patients will present respiratory tract involvement as the first sign of the illness. Although the disease is usually seen in the upper respiratory tract, various manifestations occupying a wide distribution can be found. In some studies, 8–16% of patients presented ocular manifestations as the first symptoms, but orbital manifestations were eventually present in 50–87% of patients with GPA [16]. 

In the literature, the presence of c-ANCA with a cytoplasmic staining pattern directed against proteinase 3 (PR3) is present in 80% of cases [6], resulting in the development of systemic vasculitis and granulomatous inflammation. The biopsy results support the diagnosis with evidence of necrotizing granulomas and vasculitis [23]. 

Ocular involvement isa common manifestation in patients with vasculitis. It can include tarsal-conjunctival disease, episcleritis, scleritis, keratitis, uveitis, retinal disease, nasolacrimal disease, orbital disease, and adnexal disease. The classification made by Straatsma describes the ophthalmologic involvement as continuous or non-contiguous, based on the presence or absence of direct extension from the adjacent structures [24]. 

Orbital disease and eyelid involvement may manifest as inflammation and growth of tissue from the sinuses and is commonly seen after several years of disease progression. Injury to the ocular structures can be caused by compression activity by the tumor mass, orbital cellulitis expansion, or vascular injury. The inflammation may cause optic nerve compression and ischemic optic neuropathy with visual loss [25]. Hoffman et al. reported in the NIH series that almost one half of patients with optic nerve ischemia lost vision and that 52% of patients with GPA developed an ophthalmologic disease; thus, any sign of ocular inflammation may indicate relapse of the disease in other organs [22]. 

Proptosis can be seen in 15–20% of patients with granulomatosis with polyangiitis due to orbital inflammation. It is considered an important sign since the association of proptosis with renal or lung involvement is highly suggestive for diagnosis of the disease [26]. A few cases of diplopia had been described in the literature due to the inflammation of the orbital muscles, vasculitis of the vasa vasorum, and compression of the optic nerve [27]. Supplementary, orbital fistula, or orbital abscesses due to infection can also be encountered [28,29]. 

Granulomatosis with polyangiitis can affect the lacrimal drainage system and can cause nasolacrimal obstruction and dacryoadenitis, which lead to epiphora [30]. Dacryoadenitis can be the first sign of granulomatosis with polyangiitis, as reported by Howe et al. [31], which is often bilateral [32,33], and can be suggested in a patient with pain and edema of the eyelid and anterior orbit. The dacryoadenitis may cause ocular sicca syndrome [13,22,34]. A study of 226 patients reported that 13 patients had chronic dacryocystitis due to necrotizing and destructive rhino-sinusitis [35]. 

Conjunctivitis was reported in 16% of patients in a Robinson study [11]. It can cause chronic inflammation and ulcerative conjunctivitis, which can produce cicatricial changes in the ocular surface [36,37,38]. The patients may present symptoms such as red eye, foreign body sensation, and blurred vision [13]. 

Mild episcleritis can occur [39], but scleritis is the most common ophthalmologic diagnosis in 10% of patients with granulomatosis with polyangiitis. It can cause red eye and severe pain [40]. Bullen et al., reported scleritis and episcleritis in 7 and 3.5% of patients included in their study, respectively, while retinitis was diagnosed in 2. 9% patients [13]. The involvement of the retina may vary from cotton-wool spots to intraretinal hemorrhages; occlusion of retinal and choroidal circulation has been reported in patients with vasculitis [41,42]. Impaired prognosis for visual capacity was seen in retinal manifestations [43,44]. 

Corneal involvement may also be seen in GPA, manifested as peripheral ulcerative keratitis or interstitial keratitis [45]. An immunohistochemical exam can be performed since granulomatosis with polyangiitis has a particular presentation of peripheral ulcerative keratitis (PUK)with positive antibodies to a 66-kDa corneal epithelial antigen (BCEA-A). Scleritis is a common associated presentation alongside PUK due to conjoint blood supply [46]. 

Uveitis is rarely seen as an initial manifestation of GPA [47]. A cohort study found an incidence of 17.9% of uveitis in patients with ANCA-positive vasculitis. Meanwhile, 50% of patients with anterior uveitis had coexisting scleritis, suggesting that uveitis was a secondary manifestation [45]. Granulomatous panuveitis has been described as the first manifestation of GPA [48]. 

The causes of visual loss in patients with GPA are caused by compression of the optic nerve, vasculitis of the retinal and optic nerves, and also by complications from scleritis and keratitis. Multiple factors can impact visual prognosis, such as the development of the disease and initiation of early treatment [49]. 

Studies have shown that ocular pathology can either be the first sign of GPA, or it can appear later in the development of the disease, as presented earlier. With a multidisciplinary approach to this disease, an ophthalmologic examination is required because of the possible presentation of subglottic stenosis with tarsal-conjunctival disease in the progression of GPA. 

In our case, after follow-up for 19 years, the patient presented with Sicca syndrome at the last ophthalmological examination during the COVID-19 period, for which she is receiving proper treatment. 

As for treatment of GPA, combined glucocorticoids and cyclophosphamide have generally been demonstrated to achieve remission in the majority of patients, and several trials achieved remission with the combination of methotrexate and glucocorticoids [22,50]. A randomized trial supported the advantages of cyclosporine after remission induction. The literature shows some success in remission induction with the use of immunosuppressive agents such as rituximab, infliximab, and 15-deoxyspergualin. Despite different treatment strategies [51], relapses are often encountered [18,52]. Some risk factors for relapse and treatment resistance were identified and may be used to achieve a better prognosis [53]. 

## 4. Conclusions

Granulomatosis with polyangiitis is a rare and fatal disease without treatment. Early diagnosis and treatment are key to controlling its progression. Necrotizing granulomatous inflammation and vasculitis on a biopsy, along with the presence of ANCAs, provide support for the diagnosis of the disease. Various manifestations occupying a wide distribution may be found throughout disease progression. Regular ophthalmic examination is important since a multitude of manifestations can be present, as we have seen in this case report. For the best outcome in patient treatment, a multidisciplinary approach is required.
